# Evaluating Muscle Mass Changes in Critically Ill Patients: Rehabilitation Outcomes Measured by Ultrasound and Bioelectrical Impedance

**DOI:** 10.3390/healthcare12212128

**Published:** 2024-10-25

**Authors:** Mijoo Kim, Soyun Kim, Yerin Ju, Soyoung Ahn, Song I Lee

**Affiliations:** 1 Division of Cardiology and Critical Care Medicine, Department of Internal Medicine, Chungnam National University School of Medicine, Chungnam National University Hospital, Daejeon 35015, Republic of Korea; hjmj486@cnuh.co.kr; 2 Department of Internal Medicine, Chungnam National University School of Medicine, Daejeon 35015, Republic of Korea; soyun3132@cnuh.co.kr (S.K.); jufls@cnuh.co.kr (Y.J.); 3 Division of Pulmonary and Critical Care Medicine, Department of Internal Medicine, Chungnam National University School of Medicine, Chungnam National University Hospital, Daejeon 35015, Republic of Korea; 4 Department of Rehabilitation Medicine, Chungnam National University School of Medicine, Chungnam National University Hospital, Daejeon 35015, Republic of Korea; asyoung@cnuh.co.kr

**Keywords:** breathing exercises, critically ill patients, intensive care unit, muscle mass

## Abstract

Background/Objectives: The loss of muscle mass is common in critically ill patients and is associated with poor prognosis, and efforts have been made to mitigate muscle loss through rehabilitation. This study aimed to evaluate changes in muscle mass in critically ill patients following rehabilitation. Methods: We enrolled 53 patients expected to stay in the ICU for more than 7 days, dividing them into rehabilitation (15 patients) and no rehabilitation groups (38 patients). Muscle mass was measured using ultrasound and bioelectrical impedance analysis (BIA). Results: Baseline characteristics and comorbidities showed no statistical differences between the two groups. Initial measurements of muscles showed no significant differences between the groups in rectus femoris thickness, total anterior thigh muscle thickness, cross-sectional area, echogenicity, or in-body skeletal muscle mass at baseline and 7 days. However, at 14 days, significant differences emerged. The rehabilitation group had greater rectus femoris thickness (1.42 cm vs. 0.81 cm, *p* = 0.007) and total anterior thigh muscle thickness (3.79 cm vs. 2.32 cm, *p* = 0.007) compared to the no rehabilitation group. Additionally, the rehabilitation group experienced a significantly smaller reduction in rectus femoris cross-sectional area (−4.6% vs. −22.8%, *p* = 0.021). Although survival rates were higher in the rehabilitation group (73.3% vs. 52.6%), this difference was not statistically significant (*p* = 0.096). Conclusions: Our findings suggest that rehabilitation in critically ill patients is associated with a slower rate of muscle loss, particularly in the cross-sectional area of the rectus femoris muscle, which may be beneficial for patient recovery.

## 1. Introduction

Muscle mass loss is a prevalent and significant complication among critically ill patients admitted to the intensive care unit (ICU). Possibly, ICU-acquired weakness is an umbrella term for a broad syndrome that includes not only muscle wasting but also neuromuscular dysfunction and severe functional impairment. ICU-acquired weakness is commonly observed in ICU patients and is a condition associated with prolonged immobilization, systemic inflammation, and a catabolic stress response common in the ICU [1,2]. In addition to functional impairment, muscle wasting significantly increases the risk of adverse events such as falls [3], which can have serious or fatal consequences in the hospital setting. A recent analysis [4] of Italian healthcare facilities found that only 45.6% were fully compliant with the national guidelines for fall prevention, highlighting the need for standardized strategies and improved fall management to protect vulnerable patients. This loss of muscle mass and weakness not only prevents the functional recovery of patients, but is also associated with negative outcomes, including increased mortality [5], longer ICU and hospital stays [6,7], post-ICU mortality, and decreased physical function [8].

The maintenance and restoration of muscle mass in critically ill patients have, therefore, become focal points in the management of these individuals. In this study, patients were assessed at baseline, 7 days after admission to the ICU and at discharge from the ICU, allowing us to follow changes in muscle mass over time. Rehabilitation interventions, including early mobilization, physical therapy, and structured exercise programs, are implemented according to current ICU rehabilitation protocols to prevent muscle wasting and promote recovery in critically ill patients [9,10]. While these interventions have shown potential benefits, such as improving physical function and reducing the ICU and hospital length of stay, the evidence remains inconsistent due to variability in rehabilitation modalities and challenges in measuring outcomes [11,12]. Early rehabilitation, within 72 h of ICU admission, may improve physical and cognitive function without increasing adverse events, although its impact on mental health remains uncertain. A pilot study [13] has shown that both mid-frequency and high-frequency neuromuscular electrical stimulation (NMES) have similar effects on preserving muscle thickness and contractile strength in critically ill patients, although the optimal protocol remains to be determined. Despite the theoretical benefits, evidence supporting the efficacy of these interventions remains inconsistent, partly due to challenges in accurately measuring muscle mass changes in this patient population. The traditional methods of assessing muscle mass [14,15], such as computed tomography, magnetic resonance imaging, and dual-energy X-ray absorptiometry, are accurate but impractical in the ICU due to high cost, patient transport requirements, and vital sign instability. Consequently, bedside techniques such as ultrasound [16,17] and bioelectrical impedance analysis (BIA) [18] have increasingly been studied. Ultrasound is noninvasive and effective for measuring muscle thickness and cross-sectional area, while BIA assesses muscle mass through electrical conductivity. Despite their limitations, both methods provide a comprehensive view of changes in muscle mass. Recent studies [19] have shown that BIA can effectively track muscle mass loss during ICU stays, making it useful for monitoring critically ill patients.

Considering the critical need to address muscle wasting in patients in the ICU, this study aimed to evaluate the impact of rehabilitation on muscle mass changes using ultrasound and BIA. We hypothesized that structured rehabilitation would attenuate the loss of muscle mass in critically ill patients and potentially improve survival outcomes. Specifically, we focused on the rectus femoris muscle, given its significance in mobility and overall muscle strength. Through this study, we sought to contribute to the growing body of evidence supporting early and targeted rehabilitation interventions in the ICU. In addition, rehabilitation is associated with reduced muscle wasting in critically ill patients, and we also aimed to determine the effect of early rehabilitation on muscle mass and its potential to mitigate muscle loss in critically ill patients.

## 2. Materials and Methods

### 2.1. Study Design and Population

This single-center study was conducted among patients admitted to the ICU of a university-affiliated hospital between March 2022 and November 2023. The study was approved by the Institutional Review Board of Chungnam National University Hospital (approval number: 2021-11-062). Informed written consent was obtained from each patient or their authorized representative at the time of enrollment.

Patient demographics were collected by electronic medical record review; laboratory results were collected on the day of ICU admission; Acute Physiology and Chronic Health Evaluation (APACHE) II scores and Charlson Comorbidity Index were collected on the day of ICU admission; after ICU admission, strength, walking, getting out of a chair, climbing stairs, and falling (SARC-F) scores and Katz activities of daily living (ADL) scores were collected by a questionnaire based on recent daily living experiences; ICU and hospital discharge dates and discharge status were collected for prognosis.

### 2.2. Muscle Mass Measurement

The research director and co-researcher performed ultrasound at 3 (within 48 h), 7, and 14 days after admission to the ICU; at the same time, muscle mass was measured using BIA. We used a VIVID GE E UltraEdition with B-mode imaging and a 6.5 MHz, 3.8 cm linear transducer (GE L3-12-D). Patients were positioned supine with elbows and knees in passive extension, and generous amounts of contact gel were applied to avoid muscle compression. Two landmarks were marked on each quadricep: (1) the midpoint between the anterior superior iliac spine and the superior pole of the patella, and (2) the junction of the lower third and upper two-thirds between the same points. Measurements included anterior thigh, rectus femoris echogenicity, rectus femoris cross-sectional area, rectus femoris thickness, vastus intermedius thickness, and total muscle thickness. All measurements were taken by the same examiner.

BIA was performed using an InBody S10 analyzer (Biospace, Seoul, Republic of Korea), which measures body composition by passing a weak electric current through the body. Patients were placed in the supine position while electrodes were placed on both thumbs, forefingers, and just below the ankles. This enabled measurement of muscle mass in the upper limbs and provided an indirect estimate of body composition, including fat, water, bone mass, lean body mass, and muscle mass.

### 2.3. Patients’ Rehabilitation

Patients admitted to the ICU who had muscle mass measurements were reviewed and divided into two groups based on whether or not they received rehabilitation.

The rehabilitation group received physical therapy within 48 h of hemodynamic stabilization in the ICU. The structured early ICU rehabilitation program was tailored to the patient’s level of consciousness and muscle strength and consisted of the following functional rehabilitation steps: passive joint exercises/active joint exercises/sitting exercises/standing exercises/functional exercises, such as stationary walking or higher-level activities.

The progression of these steps followed general guidelines but was adjusted at the discretion of the care team based on patient safety and tolerance. Rehabilitation also included range-of-motion exercises, strength training, balance exercises, and inspiratory muscle training. Treatment consisted of 30 min sessions twice a day, five days a week (on a regular workday), with some patients receiving 30 min PROM and AROM treatments once a day.

The no rehabilitation group received either no rehabilitation therapy or minimal bedside physical therapy that included only PROM exercises for 10 min once a day on weekdays.

### 2.4. Statistical Analysis

All continuous values were expressed as mean ± standard deviation (SD) if they followed a normal distribution or as median and interquartile range (IQR) if they did not. Categorical variables were expressed as numbers and percentages. The Mann–Whitney U test was used to compare continuous variables between two independent groups, and the Wilcoxon signed-rank test was used to examine changes over time within the same group for nonparametric data. The chi-squared test or Fisher’s exact test was used to compare categorical variables between groups. A two-tailed *p*-value ≤ 0.05 was considered statistically significant. Statistical analyses were performed with IBM SPSS version 25.0 for Windows (IBM Corp., Armonk, NY, USA). Figures were generated using GraphPad Prism version 9.0 (GraphPad Software, San Diego, CA, USA).

## 3. Results

### 3.1. Basic Characteristics of Enrolled Patients

Of the 53 participants enrolled in this study, 15 (28.3%) were in the rehabilitation group and 38 (71.6%) were in the no rehabilitation group. Table 1 shows the baseline characteristics of the patients. There were no statistical differences in age, male sex, body mass index, APACHE II score, Charlson comorbidity index, SARC-F score, Katz ADL score, or comorbidities between the two groups. Table 2 shows the laboratory findings, and none of the parameters were statistically different between the two groups.

### 3.2. Measurements of Muscle Mass over Time

Table 3 shows the changes in muscle mass parameters over time for all patients, categorized by rehabilitation status. At baseline, 53 patients were evaluated. No significant differences were found between the rehabilitation and no rehabilitation groups in rectus femoris thickness, total anterior thigh muscle thickness, cross-sectional area, echogenicity, and in-body skeletal muscle mass. After 7 days, 43 patients were re-evaluated. No significant differences were noted in any of the muscle mass parameters between the two groups. At 14 days, 15 patients were left in the study. Significant differences were observed in rectus femoris thickness (rehabilitation group: 1.42 (1.23–1.63) cm vs. no rehabilitation group: 0.81 (0.66–1.08) cm, *p* = 0.007) and total anterior thigh muscle thickness (rehabilitation group: 3.79 (3.17–5.25) vs. no rehabilitation group: 2.32 (1.90–2.80) cm, *p* = 0.007). No significant differences were found for the other parameters.

### 3.3. Changes in Muscle Mass Parameters over Time by Rehabilitation Status

**Figure 1:**
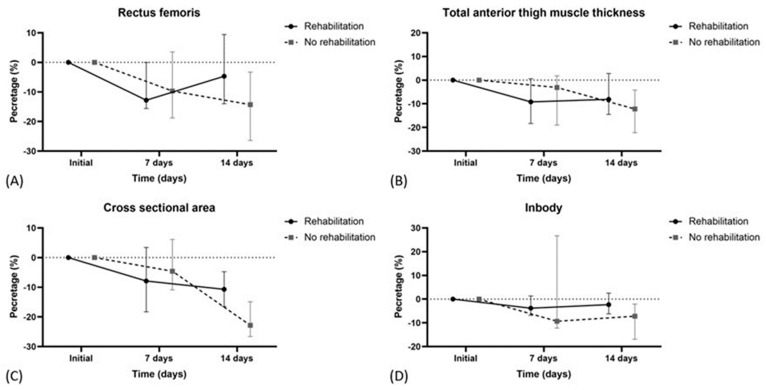
Changes in muscle mass parameters over time by rehabilitation status. (**A**) rectus femoris, (**B**) total anterior thigh muscle thickness, (**C**) cross-sectional area of the rectus femoris muscle, (**D**) in-body skeletal muscle mass.

Table 4 and Figure 1 show the percentage changes in muscle mass parameters over time for all patients categorized by rehabilitation status. At 7 days, there were no significant differences between the rehabilitation and no rehabilitation groups in rectus femoris thickness, total anterior thigh muscle thickness, or rectus femoris cross-sectional area. At 14 days, the rehabilitation group had significantly less reduction in the rectus femoris cross-sectional area (−4.6% (−10.9–6.1)) compared to the no rehabilitation group (−22.8 (−26.6–−14.9)) (*p* = 0.021). Other parameters, including echogenicity and in-body skeletal muscle mass, were not significantly different between the groups at either time point.

### 3.4. Prognosis of Patients

Table 5 shows the prognosis of the patients. The median ICU length of stay (LOS) was 10.0 days (IQR 7.0–15.0) for all patients, with no significant difference between the rehabilitation and no rehabilitation groups (11.0 (8.0–14.0) days vs. 9.5 (6.0–18.0) days, *p* = 0.542). Median in-hospital LOS was also similar between groups (17.0 (13.0–28.0) days vs. 18.5 (10.8–36.0) days, *p* = 0.931). Regarding discharge status, 58.5% of all patients survived, with higher survival in the rehabilitation group compared to the no rehabilitation group (73.3% vs. 52.6%). Discharge to the nursing care center was similar in both groups (13.3% vs. 13.2%), while mortality was lower in the rehabilitation group (13.3% vs. 34.2%). The difference in discharge outcomes was not statistically significant (*p* = 0.096).

## 4. Discussion

This study confirmed the impact of early rehabilitation on muscle mass in critically ill patients and its potential to mitigate loss. Patients in the rehabilitation group had a smaller loss of rectus femoris and anterior thigh muscle thickness at day 14 compared to those who did not receive rehabilitation. Additionally, the cross-sectional area of the rectus femoris muscle was better preserved in the rehabilitation group, highlighting the potential of structural rehabilitation to prevent muscle wasting in the ICU. These findings highlight the importance of early and sustained rehabilitation to improve muscle preservation in critically ill patients and improve overall recovery outcomes.

Our study shows results consistent with previous studies and shows the significant muscle loss observed in critically ill patients. For example, [20] reported that 59.6% of patients experienced significant muscle loss (≥10%) within 7 days. A systematic review and meta-analysis by Fazzini et al. [21] found that critically ill patients can lose nearly 2% of skeletal muscle per day during the first week of ICU admission. Their comprehensive review included 52 studies with 3251 patients and found that 55% of patients experienced significant muscle wasting and 48% developed ICU-acquired muscle weakness. This underscores the critical need for early intervention to prevent muscle mass loss during the ICU stay.

To prevent muscle weakness in these critically ill patients, early ICU rehabilitation with minimal sedation, moderate exercise, optimal respiratory muscle activation, and early enteral nutrition is recommended [22]. Several studies have evaluated the effectiveness of rehabilitation programs to preserve muscle mass in critically ill patients. Gerovasili et al. [23] demonstrated that electrical muscle stimulation (EMS) significantly preserved muscle mass and reduced cross-sectional diameter loss in the rectus femoris and vastus intermedius compared with the control group. Woo et al. [24] extended this by finding that in-bed cycling improved muscle preservation in mechanically ventilated patients, but did not show significant improvement in muscle strength when combined with EMS. Another study [25] showed that the neuromuscular electrical stimulation of muscles with active and passive exercise training further improved the prevention of muscle atrophy. Kayambu et al. [26] further investigated early physical rehabilitation in septic patients and found that early intervention resulted in a significant increase in patient self-reported physical function (81.8 ± 22.2 vs. 60.0 ± 29.4), *p* = 0.04) and physical role (61.4 ± 43.8 vs. 17.1 ± 34.4, *p* = 0.005) for the SF-36 at 6 months, which was found in the exercise group. Furthermore, a study [27] investigating early physical therapy in patients with septic shock found a significant preservation of muscle fiber cross-sectional area with intervention, with a decrease of -25.8% ± 21.6% in the control group compared to an increase of 12.4% ± 22.5% in the intervention group (*p* = 0.005). Similarly, a meta-analysis by Tipping et al. [28] found that early active exercise improved muscle strength and increased the likelihood that patients could walk unassisted at discharge, and increased survival and discharge-free days to day 180. In contrast, Rollinson et al. [29] showed that interventions such as functional electrical stimulation cycling, and supine cycling did not play a significant role in preserving muscle mass during ICU stays. Recent reviews [30] highlight the role of nutrition and physical activity in reducing muscle wasting and improving recovery after ICU admission. While randomized controlled trials (RCTs) [31] on nutrition show mixed results and no significant effect on strength or function, RCTs on physical activity suggest improved strength and function with higher levels of activity, especially if started early. Based on these studies, it is believed that incorporating regular muscle mass measurements and a comprehensive assessment program could help personalize interventions to better meet patient needs and improve outcomes.

Loss of muscle mass in critically ill patients is associated with adverse outcomes. Gruther et al. [16] found that decreased muscle mass was negatively correlated with LOS. Furthermore, skeletal muscle mass loss is associated with ICU mortality [32], 60 day mortality [17], and in-hospital mortality [33]. Additionally, a loss of muscle mass is associated with ICU-acquired weakness, which itself has significant economic implications. Early data suggest that treating patients with neuromuscular weakness is more expensive [34]. After discharge, patients face significant economic challenges [35], and many struggle to return to work due to ongoing health problems such as depression, post-traumatic stress disorder, muscle weakness, fatigue, and cognitive impairment [36]. This economic burden often forces families to make lifestyle adjustments and leads to frequent hospital re-admissions [36]. In addition, muscle wasting significantly increases the risk of falls, a common and serious complication in the hospital setting. Sarcopenia, defined as the loss of muscle mass and function, is a major risk factor for falls and fractures. A meta-analysis [3] showed that sarcopenic older adults have a significantly higher risk of falls and fractures compared to nonsarcopenic individuals. This highlights the need for appropriate interventions to preserve muscle mass, reduce the risk of falls, and improve overall outcomes in ICU patients.

Despite the findings, this study has several limitations. First, the sample size, especially in the rehabilitation group (*n* = 15), was relatively small, limiting the generalizability of the findings. However, studies with a detailed ultrasound follow-up of critically ill rehabilitation patients are rare and logistically challenging, especially in the ICU setting. Second, the single-center design may introduce selection bias and limit the external validity of our results. Third, although the prospective design allowed for structured data collection, it may have resulted in missing data and uncontrolled confounders that could affect the interpretation of the results. In addition, the small number of patients prevented us from classifying the intensity of rehabilitation each patient received, which could further influence the interpretation of the results. Finally, we did not assess long-term outcomes beyond hospitalization, which limits our understanding of the sustained benefits of rehabilitation. Future studies with larger, multicenter cohorts and more detailed data on rehabilitation intensity and long-term outcomes are needed to validate these findings and to explore the mechanisms underlying muscle preservation in critically ill patients.

## 5. Conclusions

Early rehabilitation shows promising potential for preserving muscle mass in critically ill patients, particularly in key muscle groups such as the rectus femoris. The findings are similar to previous studies that have demonstrated the benefits of structured rehabilitation interventions such as physical therapy, electrical muscle stimulation, and in-bed cycling. However, due to the limitations of this study, including the small sample size and single-center design, larger, multicenter studies would be beneficial to optimize rehabilitation protocols and implement them effectively in a variety of clinical settings to improve patient outcomes.

## Figures and Tables

**Table 1:** Baseline characteristics of enrolled patients.

Characteristics	All Patients (*n* = 53)	Rehabilitation (*n* = 15)	No Rehabilitation (*n* = 38)	*p*-Value
Age (years)	71.8 ± 11.3	71.2 ± 11.7	71.9 ± 11.3	0.601
Male	35 (66.0)	11 (73.3)	24 (63.2)	0.481
Body mass index (kg/m^2^)	22.7 ± 4.6	22.0 ± 5.0	22.9 ± 4.4	0.601
APACHE II score	20.7 ± 7.2	23.9 ± 8.4	19.5 ± 6.4	0.296
Charlson comorbidity index	2.2 ± 2.6	1.9 ± 1.6	2.3 ± 2.9	0.614
SARC-F score	3.1 ± 2.9	2.3 ± 2.5	3.5 ± 3.0	0.429
Katz ADL score	3.6 ± 2.4	3.7 ± 2.4	3.6 ± 2.4	0.995
Comorbidities				
Hypertension	31 (58.5)	6 (40.0)	25 (65.8)	0.086
Diabetes	21 (39.6)	7 (46.7)	14 (36.8)	0.510
Cerebral infarction	2 (3.8)	0 (0)	2 (5.3)	0.365
Heart failure	11 (20.8)	4 (26.7)	7 (18.4)	0.505
Liver cirrhosis	5 (9.4)	2 (13.3)	3 (7.9)	0.542
Solid tumor	12 (22.6)	3 (20.0)	9 (23.7)	0.773
Hematologic malignancy	1 (1.9)	0 (0)	1 (2.6)	0.526
COPD	12 (22.6)	5 (33.3)	7 (18.4)	0.243
Dementia	3 (5.7)	1 (6.7)	2 (5.3)	0.842

Data are presented as mean ± standard deviation or *n* (%) unless otherwise noted. APACHE: Acute Physiology and Chronic Health Evaluation. SARC-F: strength, assistance with walking, rising from a chair, climbing stairs, and falls. Katz ADL: Katz activities of daily living. COPD: Chronic obstructive pulmonary disease.

**Table 2:** Laboratory findings of enrolled patients.

Characteristics	All Patients	Rehabilitation	No Rehabilitation	*p*-Value
White blood cell, ×10^3^/µL	12.8 ± 7.5	13.5 ± 6.3	12.6 ± 8.0	0.486
Hemoglobin, g/dL	11.9 (9.7–13.7)	12.4 (10.4–14.5)	11.0 (9.5–13.4)	0.191
Platelet, ×10^3^/µL	206.9 ± 118.6	256.9 ± 128.8	187.2 ± 109.9	0.486
Total bilirubin, mg/dL	0.78 (0.50–1.20)	0.70 (0.44–1.06)	1.53 (0.58–1.47)	0.257
Albumin, g/dL	3.13 ± 0.60	3.43 ± 0.57	3.01 ± 0.58	0.384
AST, U/L	30.0 (23.5–51.5)	24.0 (18.0–52.0)	30.5 (26.0–51.3)	0.725
ALT, U/L	23.0 (15.0–42.0)	24.0 (13.0–37.0)	23.0 (15.0–43.8)	0.931
BUN, mg/dL	21.4 (16.0–40.0)	21.7 (15.8–26.3)	20.7 (16.0–46.8)	0.931
Creatinine, mg/dL	0.82 (0.62–1.56)	0.84 (0.62–1.32)	0.82 (0.62–1.81)	0.931
Na, mEq/L	137.1 ± 5.9	136.7 ± 4.9	137.2 ± 6.4	0.336
K, mEq/L	4.4 ± 0.9	4.7 ± 0.8	4.3 ± 0.9	0.486
Cl, mEq/L	102.8 ± 7.1	100.1 ± 7.0	103.9 ± 7.0	0.336
CRP, ng/mL	7.25 (1.38–8.00)	2.15 (0.10–7.63)	8.00 (3.90–9.05)	0.115
Procalcitonin, ng/mL	0.32 (0.05–2.36)	0.05 (0.05–0.28)	0.69 (0.09–4.33)	0.035
PT, INR	1.18 (1.08–1.42)	1.24 (1.04–1.36)	1.18 (1.10–1.46)	0.931
aPTT. Sec	31.2 (27.7–37.0)	31.0 (26.6–31.8)	31.8 (28.1–38.4)	0.601
Lactic acid, mEq/L	1.90 (1.30–3.05)	2.00 (1.20–3.50)	1.90 (1.38–2.98)	0.931

Data are presented as mean ± standard deviation or median and interquartile range, unless otherwise indicated. AST: aspartate transaminase. ALT: alanine transaminase. BUN: blood urea nitrogen. Na: sodium. K: potassium. Cl: chloride. CRP: C-reactive protein. PT: prothrombin time. aPTT: activated partial thromboplastin time.

**Table 3:** Measurements of muscle mass over time.

	All Patients	Rehabilitation	No Rehabilitation	*p*-Value
Initial (*n*)	53	15	36	
Rectus femoris (cm)	1.11 (0.87–1.44)	1.22 (0.85–1.51)	1.03 (0.88–1.35)	0.481
Total anterior thigh muscle thickness (cm)	2.99 (2.33–3.53)	3.33 (2.18–4.10)	2.88 (2.40–3.49)	0.481
Cross-sectional area (rectus femoris, cm^2^)	4.93 (3.95–6.19)	5.26 (4.27–6.46)	4.92 (3.81–6.07)	0.928
Echogenicity, dB	45.98 (41.13–49.65)	48.43 (43.75–50.18)	44.95 (40.18–48.86)	0.481
In body—skeletal muscle mass (kg)	24.20 (20.05–28.95)	24.70 (21.05–27.13)	24.20 (19.53–29.63)	0.753
7 days (*n*)	43	14	29	
Rectus femoris (cm)	1.03 (0.82–1.31)	1.03 (0.86–1.35)	1.03 (0.79–1.20)	0.826
Total anterior thigh muscle thickness (cm)	2.78 (2.14–3.37)	2.61 (1.85–3.93)	2.80 (2.24–3.20)	0.826
Cross-sectional area (rectus femoris, cm^2^)	4.36 (3.53–5.04)	4.54 (4.07–5.46)	4.05 (3.36–4.96)	0.326
Echogenicity, dB	44.21 (40.14–48.69)	45.86 (41.66–50.83)	44.11 (39.42–46.54)	0.743
In body—skeletal muscle mass (kg)	23.00 (19.30–25.80)	23.85 (20.03–25.65)	20.90 (18.70–26.05)	0.650
14 days (*n*)	15	5	10	
Rectus femoris (cm)	1.01 (0.72–1.32)	1.42 (1.23–1.63)	0.81 (0.66–1.08)	0.007
Total anterior thigh muscle thickness (cm)	2.78 (2.04–3.79)	3.79 (3.17–5.25)	2.32 (1.90–2.80)	0.007
Cross-sectional area (rectus femoris, cm^2^)	4.48 (3.35–5.60)	6.74 (5.04–7.23)	3.72 (2.45–4.61)	0.119
Echogenicity, dB	44.68 (42.15–47.40)	44.68 (40.43–48.08)	44.71 (42.19–49.11)	>0.999
In body—skeletal muscle mass (kg)	23.40 (21.63–28.53)	23.60 (19.50–35.88)	23.25 (21.63–25.83)	>0.999

Data are presented as median and interquartile range, unless otherwise indicated.

**Table 4:** Temporal percentage change in muscle mass from an assumed initial 100% over time.

	Initial	7 Days	*p*-Value	%Change (D7–D3)	*p*-Value	14 Days	*p*-Value	%Change (D14–D3)	*p*-Value
Rectus femoris (%)	All patients	87.9 (82.9–102.7)	0.828	−12.1 (−17.0–2.7)	0.828	89.7 (75.3–98.4)	0.119	−9.7 (−23.2–−0.5)	0.266
	Rehabilitation	87.2 (84.4–100.0)		−12.8 (−15.6–−0.02)		95.3 (86.0–109.4)		−4.7 (−14.0–9.4)	
	No rehabilitation	90.3 (81.1–103.5)		−9.7 (−18.8–3.5)		84.6 (70.6–93.8)		−14.3 (−26.4–−3.3)	
Total anterior thigh muscle thickness (%)	All patients	91.9 (85.4–102.1)	0.925	−8.1 (−14.6–2.1)	0.828	89.2 (80.9–100.3)	0.119	−11.4 (−20.6–0.6)	0.266
	Rehabilitation	89.0 (85.5–123.1)		−9.2 (−18.3–0.6)		96.9 (81.0–101.8)		−3.1 (−19.0–1.8)	
	No rehabilitation	92.1 (84.8–98.6)		−8.1 (−14.5–2.8)		88.4 (79.4–92.9)		−12.2 (−22.2–−4.2)	
Cross-sectional area (rectus femoris, %)	All patients	90.1 (82.7–98.5)	0.925	−9.9 (−17.3–−1.5)	0.828	85.2 (75.2–95.4)	0.119	−15.3 (−24.9–−6.3)	0.021
	Rehabilitation	94.1 (81.7–101.8)		−7.9 (−18.3–3.4)		95.4 (89.1–106.1)		−4.6 (−10.9–6.1)	
	No rehabilitation	89.7 (83.3–95.6)		−10.7 (−16.5–−4.74)		79.3 (74.1–88.8)		−22.8 (−26.6–−14.9)	
Echogenicity (%)	All patients	98.9 (95.2–101.6)	0.381	−1.1 (−4.8–1.6)	0.381	102.0 (99.1–103.8)	0.282	2.3 (−1.2–4.8)	0.266
	Rehabilitation	98.1 (94.7–100.6)		−1.9 (−5.2–0.6)		102.9 (100.4–110.4)		3.1 (1.3–13.1)	
	No rehabilitation	99.8 (96.0–104.4)		−0.2 (−4.0–4.4)		98.0 (93.7–101.5)		−2.0 (−6.3–1.5)	
In body—skeletal muscle mass (%)	All patients	96.2 (93.8–102.2)	0.831	−3.8 (−6.3–2.4)	0.833	91.8 (85.9–97.9)	>0.999	−8.2 (−14.0–−2.1)	>0.999
	Rehabilitation	96.2 (93.2–101.3)		−3.8 (−6.8–1.3)		90.7 (87.8–126.7)		−9.3 (−12.2–26.7)	
	No rehabilitation	97.7 (93.8–102.4)		−2.3 (−6.2–2.5)		92. 8 (83.1–97.9)		−7.2 (−16.9–−2.1)	

Data are presented as median and interquartile range, unless otherwise indicated. D: days.

**Table 5:** Prognosis of patients.

Characteristics	All Patients	Rehabilitation	No Rehabilitation	*p*-Value
ICU LOS	10.0 (7.0–15.0)	11.0 (8.0–14.0)	9.5 (6.0–18.0)	0.542
In-hospital LOS	18.0 (11.5–32.5)	17.0 (13.0–28.0)	18.5 (10.8–36.0)	0.931
State of discharge				0.096
Survival	31 (58.5)	11 (73.3)	20 (52.6)	
Nursing care center	7 (13.2)	2 (13.3)	5 (13.2)	
Death	15 (28.3)	2 (13.3)	13 (34.2)	

Data are presented as median and interquartile range or *n* (%) unless otherwise noted. ICU: intensive care unit. LOS: length of stay.

## Data Availability

All data generated or analyzed during this study are included in this published article.
